# ﻿*Sinosasagracilis* (Poaceae, Bambusoideae), a new combination supported by morphological and phylogenetic evidence

**DOI:** 10.3897/phytokeys.226.101164

**Published:** 2023-05-09

**Authors:** Xing Li, Jing-Bo Ni, Zhuo-Yu Cai, Yi-Hua Tong, Nian-He Xia

**Affiliations:** 1 Key Laboratory of Plant Resources Conservation and Sustainable Utilization & Guangdong Provincial Key Laboratory of Applied Botany, South China Botanical Garden, Chinese Academy of Sciences, CN-510650, Guangzhou, China South China Botanical Garden, Chinese Academy of Sciences Guangzhou China; 2 University of Chinese Academy of Sciences, CN-100049, Beijing, China University of Chinese Academy of Sciences Beijing China; 3 South China National Botanical Garden, CN-510650, Guangzhou, China South China National Botanical Garden Guangzhou China

**Keywords:** bamboo, phylogeny, *Sinosasa*, taxonomy

## Abstract

The results of phylogenetic analysis, based on the whole chloroplast genome and morphological study support the transfer of a long ignored bamboo species, *Sasagracilis*, to the recently established genus, *Sinosasa*, in this study. Morphologically, this species differs from all the other known *Sinosasa* species by having very short (2–3 mm) foliage leaf inner ligules, which is unusual in this genus. A revised description of its morphology and colour photos are also provided.

## ﻿Introduction

*Sinosasa* L.C.Chia ex N.H.Xia, Q.M.Qin & Y.H.Tong was recently segregated from *Sasa*Makino and Shibata (1901) to accommodate some species previously placed in Sasasubg.Sasa from China, based on morphological and phylogenetic evidence (Qin et al. 2021). This genus differs from *Sasa* in having raceme-like (vs. panicle-like) synflorescences, two to three (vs. four to ten) florets per spikelet with a rudimentary terminal floret, three (vs. six) stamens and two (vs. three) stigmas per floret, wavy (vs. usually flat) foliage leaf blades when dry and relatively long (> 1 cm) (vs. short) foliage leaf inner ligules (Qin et al. 2021). Up to now, *Sinosasa* contains seven species endemic to subtropical areas of China and usually found growing along the river valley or in moist areas under evergreen broad-leaved forests at elevations of 700–1200 m (Qin et al. 2021).

*Sasagracilis* B.M.Yang (1990) was described based on the only collection *B. M. Yang 06774* from Shangmuyuan, Jiangyong County, Hunan Province, China. After its publication, it is only recognised in ‘Bamboos of Hunan’ (Yang 1993), edited by the author of this name, ‘Iconographia Bambusoidearum Sinicarum’ (Yi et al. 2008) and its English version ‘Illustrated Flora of Bambusoideae in China’ (Shi et al. 2022). However, because of the narrow circulation of the publication Acta Scientiarum Naturalium Universtis Normalis Hunanensis (later the name was changed to Journal of Natural Science of Hunan Normal University) at that time in China (Deng and Xia 2014), this species was ignored by the widely distributed monographs, such as ‘Flora Reipublicae Popularis Sinicae’, ‘Flora of China’, ‘World Checklist of Bamboos and Rattans’ (Hu 1996; Wang and Stapleton 2006; Vorontsova et al. 2016), the well-known database GrassBase-The Online World Grass Flora (Clayton et al. 2016) and some important websites like http://www.ipni.org, http://www.tropicos.org and http://www.theplantlist.org. In the protologue, this species was described to possess a suite of vegetative characters, such as solitary branches at each branching node, strongly raised supranodal ridges and wavy foliage leaf blades when dry, which fit well with the circumscription of *Sinosasa*. However, this species has very short foliage leaf inner ligules that are only 2–3 mm long, while all hitherto known *Sinosasa* species typically have more than 1 cm long inner ligules. Therefore, the taxonomic position of *Sasagracilis* needs a further study.

## ﻿Materials and methods

The specimens of *Sasagracilis* were collected from its type locality during a field trip in September 2022. Fresh foliage leaves were deposited in silica gel for DNA extraction. Type specimens of *Sasagracilis* deposited in the Herbarium of Hunan Normal University (HNNU) were examined. Observations and measurements were taken using a magnifier and a ruler with a scale of 0.5 mm. Some minor characters like the indumentum were observed with a stereomicroscope (Mshot MZ101). The morphological terms follow McClure (1966) and Beentje (2016). Herbarium acronyms follow Thiers (2022, continuously updated).

To study the phylogenetic position of *Sasagracilis* within the tribe Arundinarieae, the whole chloroplast genomes were used for building the phylogenetic tree. A total of 24 representatives belonging to all the five subtribes of the tribe Arundinarieae (Zhang et al. 2020a) were sampled and *Bambusamultiplex* (Lour.) Raeusch. ex Schult. f. from the tribe Bambuseae was used as outgroup. All the sampled taxa, as well as their voucher information and GenBank accession numbers, are listed in Table 1.

**Table 1.** List of 25 bamboo taxa sampled in the present study with the related voucher and GenBank accession information.

Taxon	Voucher information	Accession number
**Ingroup**		
*Acidosasaglauca* B.M.Yang	CZY56 (IBSC)	OP850353
*Ampelocalamusactinotrichus* (Merr. & Chun) S.L.Chen, T.H.Wen & G.Y.Sheng	MPF10003 (KUN)	MF066245
*Chimonobambusatumidissinoda* Ohrnb.	MPF10083 (KUN)	MF066244
*Fargesiaedulis* Hsueh f. & T.P.Yi	D418 (SANU)	MH988735
*Gaoligongshaniamegalothyrsa* (Hand.-Mazz.) D.Z.Li, Hsueh & N.H.Xia	MPF10056 (KUN)	JX513419
*Gelidocalamusstellatus* T H.Wen	BH102 (IBSC)	OP850347
*Hsuehochloacalcareus* (C.D.Chu & C.S.Chao) D.Z.Li & Y.X.Zhang	MPF10050 (KUN)	KJ496369
*Indocalamuslongiauritus* Hand.-Mazz.	MPF10168 (KUN)	HQ337795
*Indocalamussinicus* (Hance) Nakai	ZMY037 (KUN)	MF066250
*Indosasacrassiflora* McClure	BH58 (IBSC)	OK558536
*Oligostachyumsulcatum* Z.P.Wang & G.H.Ye	Not provided by the author	MW190089
*Phyllostachysedulis* (Carriere) J.Houzeau	MPF10163 (KUN)	HQ337796
*Pleioblastusmaculatus* (McClure) C.D.Chu & C.S.Chao	MPF10161 (KUN)	JX513424
*Pseudosasacantorii* (Munro) Keng f.	MPF10006 (KUN)	MF066255
*Pseudosasajaponica* (Siebold & Zucc. ex Steud.) Makino ex Nakai	Pjc-1 (ZJFC)	KT428377
*Ravenochloawilsonii* (Rendle) D.Z.Li & Y.X.Zhang	MPF10146 (KUN)	JX513421
*Sasaveitchii* Rehder	LC1325 (ISC)	KU569975
*Sasagracilis* B.M.Yang	LX153 (IBSC)	*OP973764
*Shibataeachiangshanensis* T.H.Wen	ZLN-2011080 (KUN)	MF066257
*Sinosasafanjingshanensis* N.H.Xia, Q.M.Qin & J.B.Ni	BH124 (IBSC)	OP850348
*Sinosasalongiligulata* (McClure) N.H.Xia, Q.M.Qin & J.B.Ni	CZY163 (IBSC)	OP850351
*Sinosasa* sp.	CZY173 (IBSC)	OP850352
*Sinobambusatootsik* (Makino) Makino ex Nakai	NH031 (IBSC)	OP850357
*Yushanianiitakayamensis* (Hayata) Keng f.	Not provided by the author	MN310560
**Outgroup**		
*Bambusamultiplex* (Lour.) Raeuschel ex Schult. & Schult. f.	Not provided by the author	KJ722536

### ﻿DNA extraction, sequencing, assembly and annotation

Total genomic DNA was extracted from leaves dried in silica gel using the Plant Genomic DNA Kit and then sent to Novogene (Tianjin, China) for DNA quality assessment. The qualified DNA fragments with 350 bp insert size were enriched by PCR experiment. Paired reads were sequenced on an Illumina NovaSeq 6000 platform. A total of 40 G genome skimming data (150 bp read length) were generated for each sample. These sequenced data were used to assemble the whole chloroplast genome by GetOrganelle v. 1.7.4 pipeline (Jin et al. 2018) using *Phyllostachysedulis* (GenBank accession number: HQ337796) set as the reference, with k-mer values of 45, 65, 85, 105 and 125. The complete assembly graph was visualised for plastid contig in Bandage software (Wick et al. 2015). Finally, the sequence editing was manually operated in Geneious v. 9.1.4 (Kearse et al. 2012) with the structure of LSC-IRa-SSC-IRb.

### ﻿Phylogenetic analysis

All the whole chloroplast genomes were aligned with MAFFT v. 7.490 (Katoh and Standley 2013) and combined as a data matrix. Phylogenetic analyses were conducted using Maximum Likelihood (ML) and Bayesian Inference (BI) implemented in the PhyloSuite v.1.2.2 platform (Zhang et al. 2020b). The best substitution model (K81 + GTR) for the combined data was determined using the Bayesian Information Criterion (BIC) in ModelFinder (Kalyaanamoorthy et al. 2017). A standard Maximum Likelihood tree search was performed using IQ-TREE v.1.6.8 (Nguyen et al. 2015). Nodal support (bootstrap support; BS) was assessed using 1000 standard bootstrap replicates. Bayesian phylogenetic inference was performed using MrBayes v.3.2.6 (Ronquist et al. 2012). Posterior Probability (PP) was obtained from Metropolis-coupled Markov Chain Monte Carlo simulations (two independent runs; four chains; 40,000,000 generations, sampling frequency of once every 4000 generations; 25% burn-in). Visualisation of ML and BI trees was done in FigTree v. 1.4.4 (Rambaut 2018).

## ﻿Result

**Figure 1.**
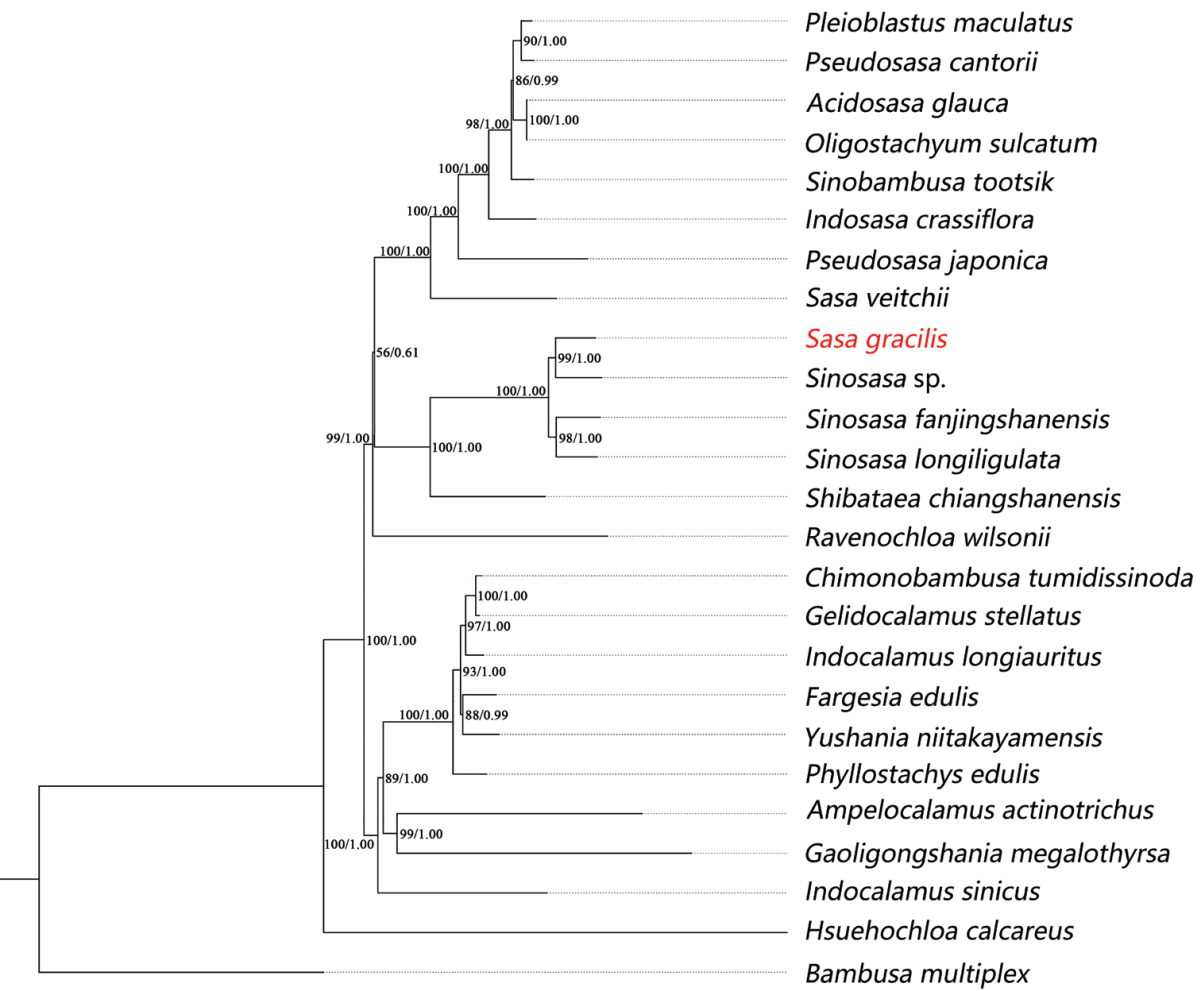
Phylogenetic tree reconstruction for *Sasagracilis*, based on plastid genome dataset with Maximum Likelihood and Bayesian analyses. Bootstrap values and posterior probabilities are indicated at each node.

The chloroplast genome size of *Sasagracilis* is 140,013 bp and those of all the samples ranged from 139,394 bp (*Bambusamultiplex*) to 140,064 bp (*Gaoligongshaniamegalothyrsa* (Hand.-Mazz.) D.Z.Li, Hsueh & N.H.Xia) with an alignment of 144,169 bp. The data matrix was characterised by sequence divergence with 3,688 variable sites (2.56%), including 773 parsimony informative sites (0.54%) and 2,915 singleton variable sites (2.02%). The phylogenetic trees, generated by the ML and BI methods, were generally consistent in topology, so only the ML tree was shown with nodal support values from both methods labelled on each node (Fig. 1). As shown in the phylogenetic tree, *Sasagracilis* is distantly related to *Sasaveitchii* Rehder (= *Sasaalbomarginata* (Miq.) Makino & Shibata, the type of *Sasa*), but forms a monophyletic clade with three *Sinosasa* species with strong nodal support (BS = 100% and PP = 1.00).

## ﻿Discussion

Our phylogenetic analysis and previous studies of Zeng et al. (2010), Zhang et al. (2012), Guo et al. (2021) and Qin et al. (2021) all demonstrated that *Sinosasa* is monophyletic. Molecular evidence, based on plastid genomic data, further confirmed that *Sasagracilis* should be a member of *Sinosasa* rather than *Sasa*. Morphologically, the characters of this species also match well with those of *Sinosasa* species as mentioned above. The short inner ligule (2–3 mm) of *Sasagracilis* can easily differentiate this species from all the other *Sinosasa* species. Consequently, the previous circumscription of *Sinosasa* on the length of foliage leaf inner ligule should be modified. In other words, *Sinosasa* does not always have long (typically > 1 cm) foliage leaf inner ligules as we knew previously. If this character is excluded, *Sasagracilis* is somewhat similar to two other *Sinosasa* species also with culm leaf auricles, viz. *Sinosasamagninoda* (T.H.Wen & G.L.Liao) N.H.Xia, Q.M.Qin & X.R.Zheng and *Sinosasaguangxiensis* (C.D.Chu & C.S.Chao) N.H.Xia, Q.M.Qin & X.R.Zheng. It further differs from *Sinosasaguangxiensis* by the glabrous (vs. densely strigose) abaxially mid- and upper part of the culm leaf sheath with short (0.5–1 mm vs. 1–3 mm) ligules and the glabrous (vs. puberulent) foliage leaf sheath and from *Sinosasamagninoda* by having culm leaf with shorter (0.5–1 mm vs. 1–3 mm) ligules, larger (2–4 × 1–2 mm vs. 1–1.5 × 1 mm) auricles and (vs. absent) oral setae and foliage leaf with more (7–12 vs. 1–4) and longer (8–20 mm vs. 5–10 mm) oral setae. A more detailed comparison amongst the three species is provided in Table 2. Based on the above evidence, it is concluded that *Sasagracilis* represents a distinct species of *Sinosasa* and a new combination of it under *Sinosasa* should be made.

**Table 2.** Comparison of *Sasagracilis*, *Sinosasaguangxiensis* and *Sinosasamagninoda*.

Characters	*Sasagracilis*	*Sinosasaguangxiensis*	*Sinosasamagninoda*
Culm leaf			
Sheath	Glabrous on the mid- and upper part	Densely strigose	Glabrous or sparsely strigose
Auricles	Elliptic to falcate, 2–4 ´ 1–2 mm	Ovate or oblong, 2–3 ´ 1–2 mm	Ovate, 1–1.5 × 1 mm
Oral setae	Present, 2–10 mm long	Present, 3–10 mm long	Absent
Ligule	0.5–1 mm	1–3 mm	1–3 mm
Foliage leaf			
Sheath	Glabrous	Puberulent	Glabrous
Oral setae	7–12, 8–20 mm	Ca. 10, 6–10 mm long	1–4, 5–10 mm
Inner ligule	2–3 mm	10–15 mm	8–12 mm

## ﻿Taxonomic treatment

### ﻿ Sinosasa gracilis

Taxon classificationPlantaePoalesPoaceae

(B.M.Yang) N.H.Xia, Y.H.Tong, J.B.Ni & X. Li comb. nov.

37E0886A-5D34-5416-89A2-38BD7AEE27FC

urn:lsid:ipni.org:names:77318864-1

Figs 2 , 3

#### Basionym.

*Sasagracilis* B. M. Yang, Acta Sci. Nat. Univ. Norm. Hunan. 13 (supplement): 1 (1990).

#### Type.

China. Hunan: Jiangyong County, Dayuan Township, Shangmuyuan, elev. 620 m, 28 October 1987, *B. M. Yang 6774* (Holotype: HNNU00053209!)

#### Description.

Shrubby bamboos. Rhizomes leptomorph, internodes 4–5.5 cm long, 2–3.5 mm in diameter, solid. Culms pluricaespitose, 0.2–1.5 m tall, 1.5–7 mm in diameter; internodes terete, 2–15 cm long, upper part initially densely white puberulent, glabrescent when old, hollow; supranodal ridge 5–9 mm in diameter, strongly raised; intranode ca. 3 mm long, glabrous, densely puberulous in infranodal region; branches extravaginal, solitary at each branching node. Culm bud solitary, trullate, sunken into supranodal ridge, ciliate on the margin. Culm leaf sheath persistent or tardily deciduous, 1/2–1/3 as long as internodes, glabrous abaxially, except for the base with a 2–3 mm wide brown and downward-appressed hispid band, glabrous on the margin; sheath scar flat or slightly prominent; auricles elliptic to falcate, 2–4 × 1–2 mm, oral setae curved, 2–10 mm long; ligule truncate, 0.5–1 mm high, ciliolate on the margin; blade triangular to lanceolate, erect or reflexed, 5–10 mm long, glabrous, serrulate along the margin. Foliage leaves 5–12 per ultimate branch; sheath glabrous, green or purple-green, 8–12 mm long, margin glabrous, longitudinal ribs conspicuous; auricles elliptic to falcate, 1–2 × 1–1.5 mm, oral setae developed, 7–12, erect or curled, 8–20 mm long; inner ligule 2–3 mm high, slightly arcuate to truncate, glabrous; outer ligule ca. 1 mm high, abaxially puberulous, white ciliate on the margin; pseudopetioles glabrous, 4–8 mm long; blades oblong-lanceolate to lanceolate, 7–29 × 1.5–5 cm, papery, wavy when dry, both surfaces glabrous, apex long-attenuate, base cuneate to obtuse, margin serrulate; secondary veins 3–8 pairs, transverse veins conspicuous. Inflorescence unknown.

**Figure 2.**
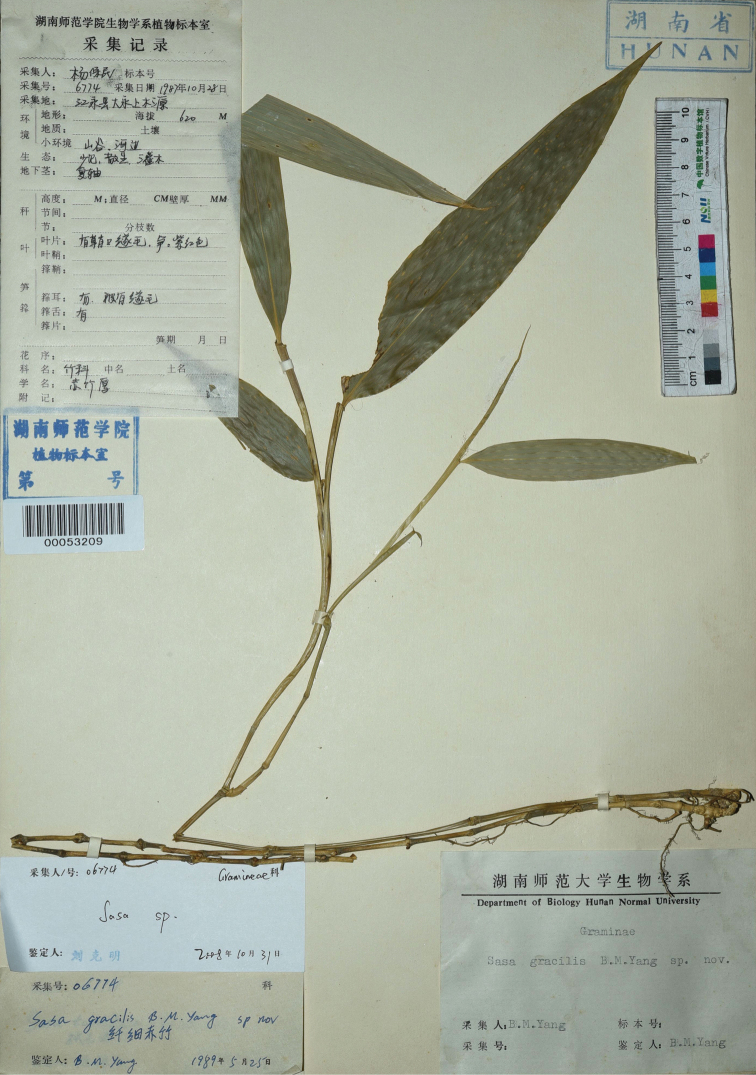
Holotype of *Sinosasagracilis* (B.M.Yang) N.H.Xia, Y.H.Tong, J.B.Ni & X.Li (≡ *Sasagracilis* B.M.Yang), *B. M. Yang 06774* (HNNU, barcode: 00053209). Photo by Zhuo-Yu Cai.

**Figure 3.**
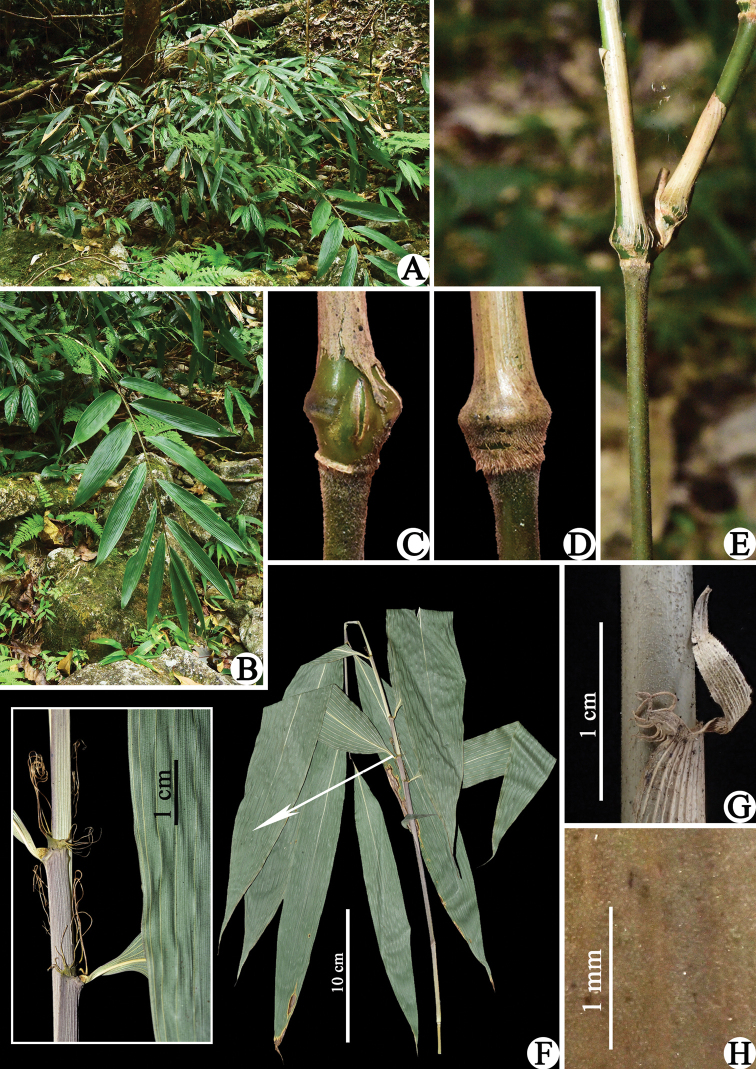
*Sinosasagracilis***A** habit **B** foliage leafy branch **C** culm bud and strongly raised supranodal ridge **D** culm leaf sheath base **E** partial culm, showing solitary branch **F** dried foliage leafy branch, showing wavy blades and a close-up view of glabrous sheath, short inner ligules, auricles and oral setae **G** culm leaf blade, auricles and oral setae **H** glabrous abaxial surface of culm leaf sheath. All photos by Xing Li.

#### Phenology.

New shoots from April to May.

#### Distribution and habitat.

It is endemic to Shangmuyuan Mountain in Jiangyong County, Hunan, China. It grows in moist places along the river banks in the valley at elevations of 600–1000 m.

#### Chinese name.

纤细华赤竹 (Chinese pronunciation: xiān xì huá chì zhú).

#### Additional specimens examined.

*Sinosasagracilis*: China. Hunan: Jiangyong County, Dayuan Township, Shangmuyuan, 18 September 2022, 25°24'24.7"N, 111°16'17.9"E, elev. 838 m, *X. Li & J. B. Ni LX153* (IBSC).

*Sinosasaguangxiensis*: China. Guangxi: Rongshui County, Jiuwan Mountain, Gema, elev. 800 m, 23 April 1979, *C. D. Chu & Z. Wang 7906* (isotypes: PE0008644, image, N019023145, image); Lingchuan County, Dajing Township, Qifen Mountain, 30 July 2006, *C. X. Zeng & Y. X. Zhang 06179* (KUN0720003, image, KUN0719374, image, KUN0719386, image, KUN0719166, image, KUN0719167, image, KUN0719168, image, KUN0719169, image).

*Sinosasamagninoda*: China. Jiangxi: Jinggang Mountain, Longtan, Zhenzhutan, 26 May 1985, *Liao et Xu 85017* (ZJFI); ibid. 28 May 1990, elev. 700 m, *T. H. Wen & G. L. Liao 90551* (Holotype: ZJFI); ibid. 27 Aug 2017, *X. R. Zheng 25* (IBSC).

## Supplementary Material

XML Treatment for Sinosasa gracilis
